# The genome sequence of a flea beetle, *Neocrepidodera transversa *(Marsham, 1802)

**DOI:** 10.12688/wellcomeopenres.23697.1

**Published:** 2025-02-10

**Authors:** Michael F. Geiser, Ian Sims, Natural History Museum Genome Acquisition Lab, Darwin Tree of Life Barcoding collective, Wellcome Sanger Institute Tree of Life Management, Samples and Laboratory team, Wellcome Sanger Institute Scientific Operations: Sequencing Operations, Wellcome Sanger Institute Tree of Life Core Informatics tea, Tree of Life Core Informatics collective, Darwin Tree of Life Consortium

**Affiliations:** 1 Natural History Museum, London, England, UK; 2 Syngenta International Research Station, Jealott’s Hill, Berkshire, England, UK

**Keywords:** Neocrepidodera transversa, flea beetle, genome sequence, chromosomal, Coleoptera

## Abstract

We present a genome assembly from a male specimen of *Neocrepidodera transversa* (flea beetle; Arthropoda; Insecta; Coleoptera; Chrysomelidae). The genome sequence has a total length of 671.30 megabases. Most of the assembly (93.58%) is scaffolded into 21 chromosomal pseudomolecules, including the X chromosome. The mitochondrial genome has also been assembled and is 17.02 kilobases in length. Gene annotation of this assembly on Ensembl identified 13,840 protein-coding genes.

## Species taxonomy

Eukaryota; Opisthokonta; Metazoa; Eumetazoa; Bilateria; Protostomia; Ecdysozoa; Panarthropoda; Arthropoda; Mandibulata; Pancrustacea; Hexapoda; Insecta; Dicondylia; Pterygota; Neoptera; Endopterygota; Coleoptera; Polyphaga; Cucujiformia; Chrysomeloidea; Chrysomelidae; Galerucinae; Alticini; *Neocrepidodera*; *Neocrepidodera transversa* (Marsham, 1802) (NCBI:txid877844)

## Background

*Neocrepidodera transversa* (Marsham, 1802) is one of over 127 British species of flea beetles ( Duff, 2018). Traditionally, flea beetles are classified as Alticinae, a separate subfamily of leaf beetles (Chrysomelidae). More recently, however, they are usually treated as a tribe within the subfamily Galerucinae, due to their close phylogenetic relationship ( Douglas *et al.*, 2023; Nie *et al.*, 2017). The genus *Neocrepidodera* Heikertinger, 1911, previously known as *Asiorestia* Jakobson, 1925, is native to the Palaearctic, Nearctic and Oriental regions. At present, there are about 80 described species, of which 66 occur in the Palaearctic Region ( Bezděk & Sekerka, 2024) and only three in North America ( Konstantinov, 1995; LeSage & Savard, 2012). Within the British fauna, three species are recorded: *N. transversa*, *N. ferruginea* (Scopoli, 1763) and *N. impressa* (Fabricius, 1801). This is the first report of a full genome for a member of this genus.

The three British *Neocrepidodera* species are all orange to rusty red flea beetles of above average size (2.6–5.5 mm), distinguished from other flea beetle genera by the combination of the following characters: 1. Pronotum with an impressed transverse groove near the base, which is delimited on each side by a little fold, connecting the basal margin to the groove at a right angle (similar to the pronotum in *Crepidodera* Chevrolat, 1836 and *Derocrepis* Weise, 1886). 2. Frontal tubercules without a clearly delimited boundary towards the vertex of the head. 3. Unlike in other Alticini with similar colour (e.g. *Sphaeroderma* Stephens, 1831 and some *Longitarsus* Latreille, 1829), the elytral punctures are clearly visible even at lower magnification and arranged into more or less regular puncture rows. These elytral puncture rows are also a good taxonomic character at the species level, being deeply impressed and completely regular in *N. ferruginea*, shallower and less regular, often doubled in the basal half, in *N. transversa* and *N. impressa*. Finally, *N. transversa* is distinguished from the similar *N. impressa* by the finely punctate pronotum (almost impunctate in *N. impressa*) with a less curved and less deeply impressed transverse groove. *N. transversa* normally ranges from 4–5 mm, but small males of only 3.5 mm length are known ( Duff, 2016; Warchałowski, 2010).

*N. transversa* is a widespread western Palaearctic species, found in all parts of Europe except northern Scandinavia and Iceland, spreading into Anatolia, Cyprus, Azerbaijan and Iran ( Bezděk & Sekerka, 2024). Within the UK, it is one of the most common and widespread species of Alticini, found virtually everywhere in England, Wales, Scotland and Northern Ireland, only absent from Orkney and Shetland ( Cox, 2007; NBN Atlas Partnership, 2024).

Adults of *N. transversa* typically occur rather late in the season, from late June to October, although some have been found as early as April ( Cox, 2007). They occur in a large variety of habitats, preferring open and somewhat wet areas, including fens, saltmarshes, agricultural lands, parks and gardens ( Cox, 2007; authors’ own observations). Adults have been found feeding on a wide range of plant families, but thistles of the genus *Cirsium* often seem to be preferred ( Rheinheimer & Hassler, 2018).

Surprisingly, the larva of this common beetle is still undescribed, and its biology and host plant association are still subject to speculation. Most likely, the larvae are root feeders, with both thistles and grasses suspected as potential hosts ( Rheinheimer & Hassler, 2018). Unlike in other flea beetles, the adults do not usually survive into winter and the larvae are suspected to overwinter ( Cox, 2007; Rheinheimer & Hassler, 2018).

**Figure 1.**
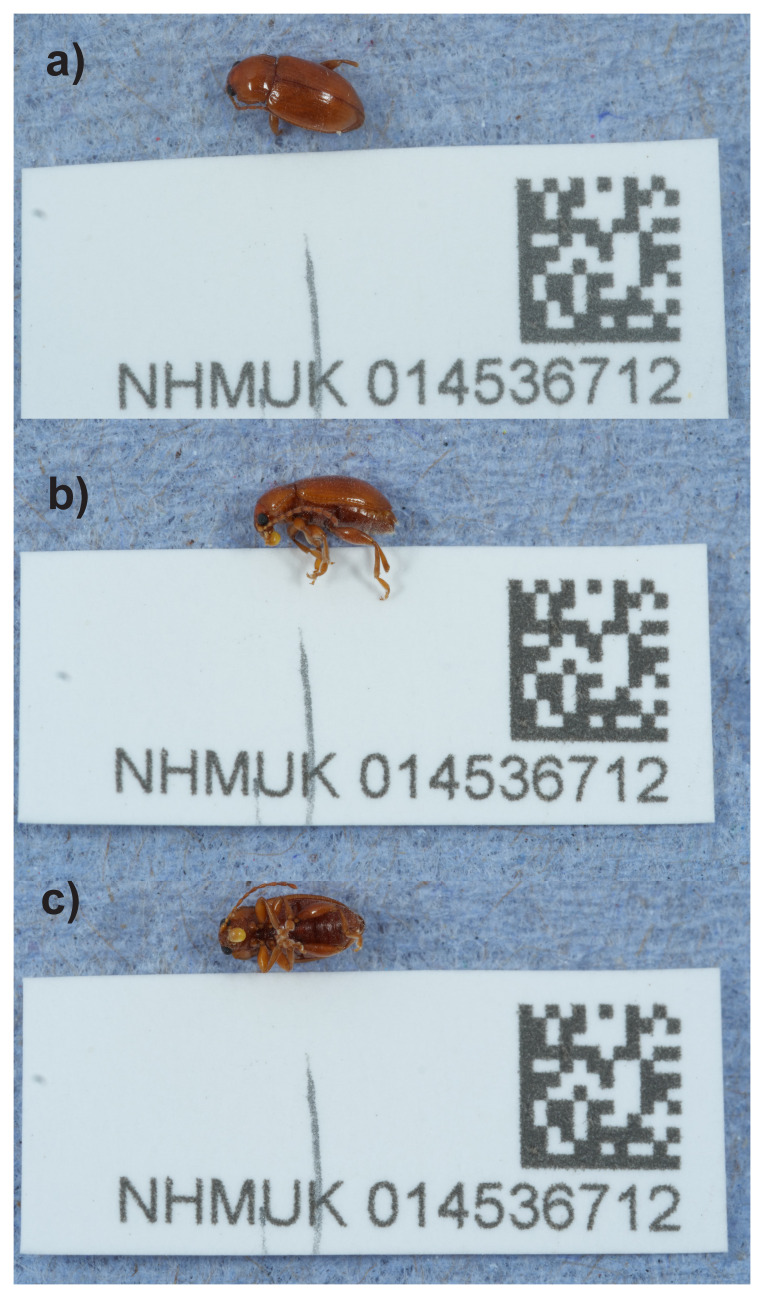
Photographs of the *Neocrepidodera transversa* (icNeoTran1) specimen used for genome sequencing.

We present a chromosomally complete genome sequence for *Neocrepidodera transversa*, based on a specimen from Hartslock Nature Reserve, England, United Kingdom ( Figure 1).

## Genome sequence report

The genome of *Neocrepidodera transversa* ( Figure 1) was sequenced using Pacific Biosciences single-molecule HiFi long reads, generating a total of 24.65 Gb (gigabases) from 1.98 million reads, providing an estimated 34-fold coverage. Chromosome conformation Hi-C sequencing produced 167.53 Gb from 1,109.44 million reads. Specimen and sequencing details are summarised in Table 1.

**Table 1.** Specimen and sequencing data for *Neocrepidodera transversa*.

Project information			
**Study title**	Neocrepidodera transversa		
**Umbrella BioProject**	PRJEB59946		
**Species**	*Neocrepidodera transversa*		
**BioSample**	SAMEA111458439		
**NCBI taxonomy ID**	877844		
Specimen information			
**Technology**	**ToLID**	**BioSample ** **accession**	**Organism part**
**PacBio long read sequencing**	icNeoTran1	SAMEA111458493	abdomen
**Hi-C sequencing**	icNeoTran1	SAMEA111458539	Head and thorax
**RNA sequencing**	icNeoTran2	SAMEA112222127	Whole organism
Sequencing information			
**Platform**	**Run accession**	**Read count**	**Base count (Gb)**
**Hi-C Illumina NovaSeq 6000**	ERR10908621	1.11e+09	167.53
**PacBio Sequel IIe**	ERR10906092	1.98e+06	24.65
**RNA Illumina NovaSeq 6000**	ERR11837468	6.62e+07	10.0

Assembly errors were corrected by manual curation, including 47 missing joins or mis-joins and seven haplotypic duplications. This reduced the assembly length by 1.03% and the scaffold number by 11.56%, and increased the scaffold N50 by 0.7%. The final assembly has a total length of 671.30 Mb in 282 sequence scaffolds, with 186 gaps, and a scaffold N50 of 29.3 Mb ( Table 2).

**Table 2.** Genome assembly data for *Neocrepidodera transversa*, icNeoTran1.1.

Genome assembly		
Assembly name	icNeoTran1.1	
Assembly accession	GCA_963243735.1	
*Accession of alternate haplotype*	*GCA_963277525.1*	
Span (Mb)	671.30	
Number of contigs	469	
Number of scaffolds	282	
Longest scaffold (Mb)	41.73	
Assembly metrics *		*Benchmark*
Contig N50 length (Mb)	5.9	*≥ 1 Mb*
Scaffold N50 length (Mb)	29.3	*= chromosome N50*
Consensus quality (QV)	60.3	*≥ 40*
*k*-mer completeness	98.05% (combined)	*≥ 95%*
BUSCO v5.4.3 lineage: endopterygota_odb10	C:98.9%[S:97.5%,D:1.4%], F:0.2%,M:0.9%,n:2,124	*S > 90%*, *D < 5%*
Percentage of assembly mapped to chromosomes	93.58%	*≥ 90%*
Sex chromosomes	X	*localised homologous pairs*
Organelles	Mitochondrial genome: 17.02 kb	*complete single alleles*
Genome annotation of assembly GCA_963243735.1 at Ensembl		
Number of protein-coding genes	13,840	
Number of non-coding genes	1,881	
Number of gene transcripts	22,975	

* Assembly metric benchmarks are adapted from Rhie *et al.* (2021) and the Earth BioGenome Project Report on Assembly Standards September 2024.

**Figure 2.**
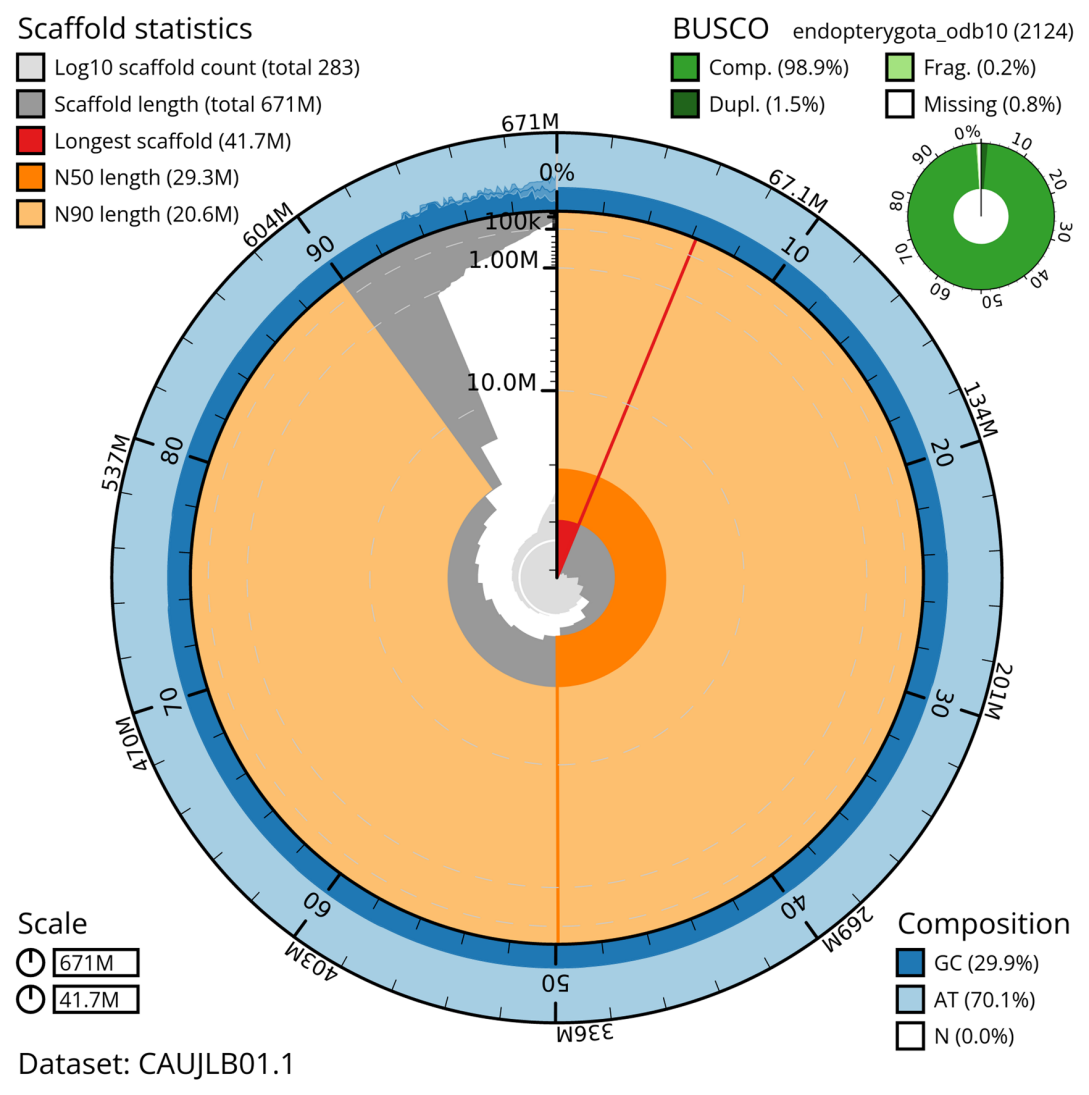
Genome assembly of *Neocrepidodera transversa*, icNeoTran1.1: metrics. The BlobToolKit snail plot provides an overview of assembly metrics and BUSCO gene completeness. The circumference represents the length of the whole genome sequence, and the main plot is divided into 1,000 bins around the circumference. The outermost blue tracks display the distribution of GC, AT, and N percentages across the bins. Scaffolds are arranged clockwise from longest to shortest and are depicted in dark grey. The longest scaffold is indicated by the red arc, and the deeper orange and pale orange arcs represent the N50 and N90 lengths. A light grey spiral at the centre shows the cumulative scaffold count on a logarithmic scale. A summary of complete, fragmented, duplicated, and missing BUSCO genes in the endopterygota_odb10 set is presented at the top right. An interactive version of this figure is available at https://blobtoolkit.genomehubs.org/view/CAUJLB01.1/dataset/CAUJLB01.1/snail.

**Figure 3.**
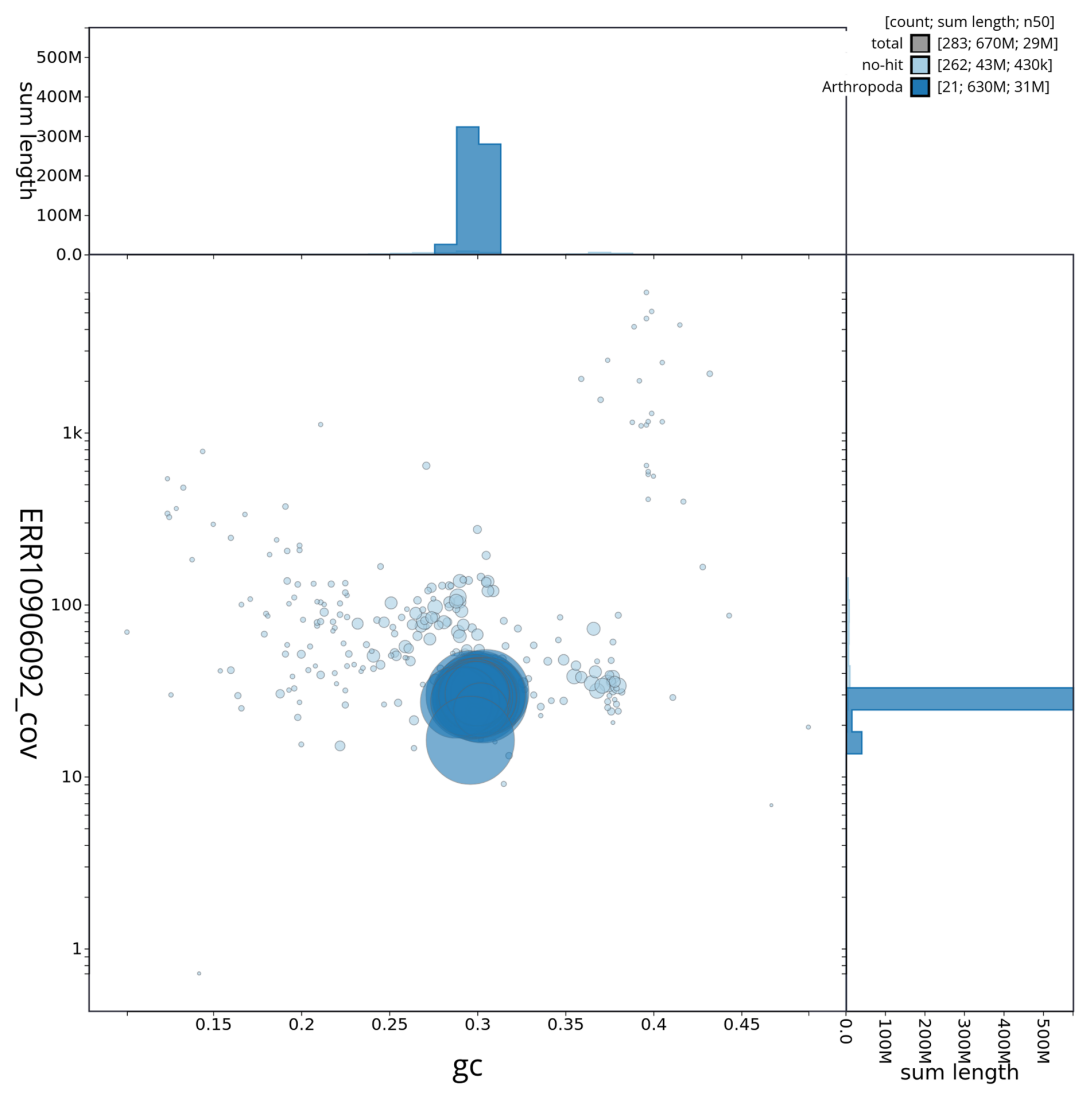
Genome assembly of *Neocrepidodera transversa*, icNeoTran1.1: BlobToolKit GC-coverage plot showing sequence coverage (vertical axis) and GC content (horizontal axis). The circles represent scaffolds, with the size proportional to scaffold length and the colour representing phylum membership. The histograms along the axes display the total length of sequences distributed across different levels of coverage and GC content. An interactive version of this figure is available at https://blobtoolkit.genomehubs.org/view/CAUJLB01.1/dataset/CAUJLB01.1/blob.

**Figure 4.**
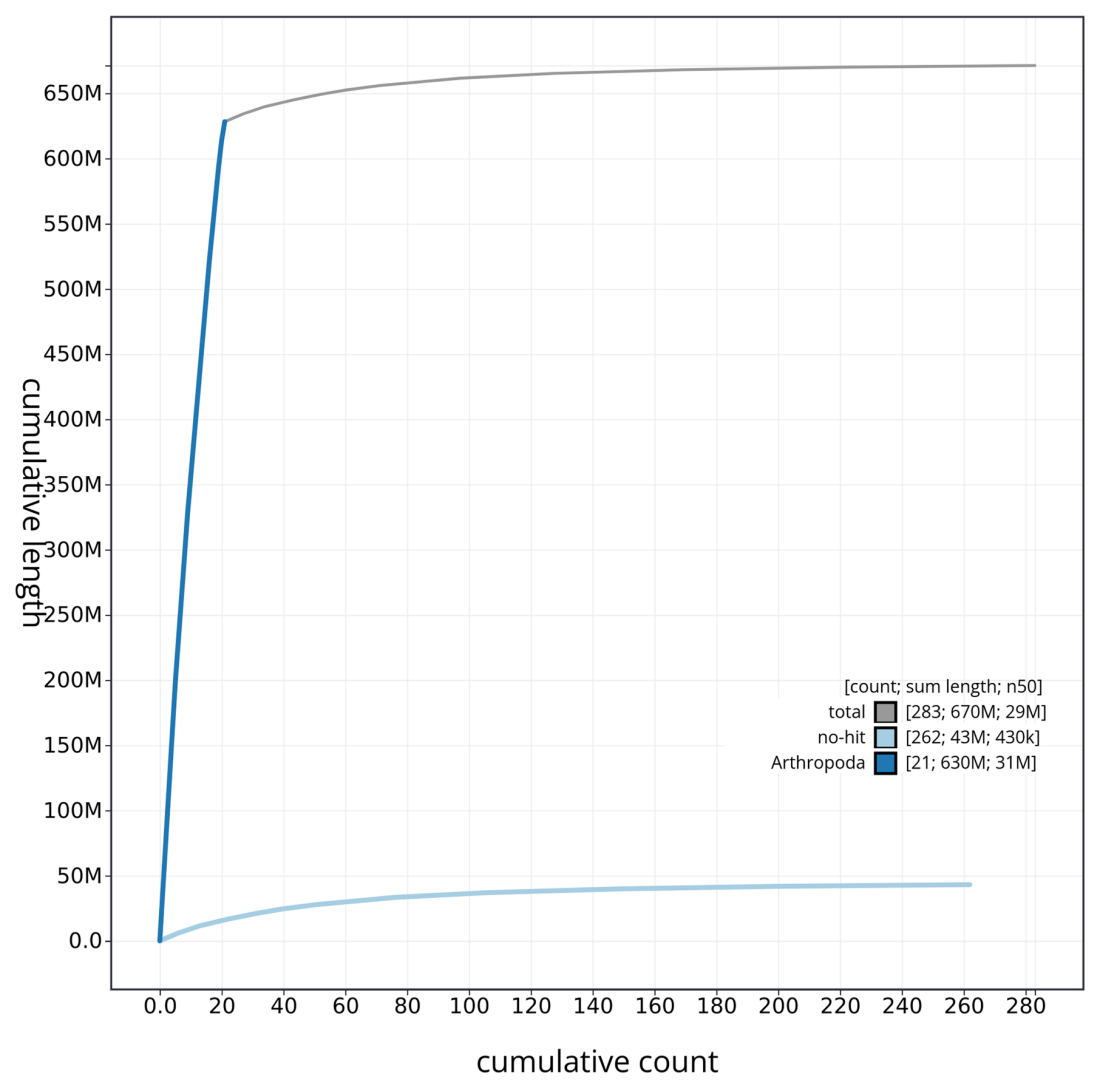
Genome assembly of *Neocrepidodera transversa* icNeoTran1.1: BlobToolKit cumulative sequence plot. The grey line shows cumulative length for all sequences. Coloured lines show cumulative lengths of sequences assigned to each phylum using the buscogenes taxrule. An interactive version of this figure is available at https://blobtoolkit.genomehubs.org/view/CAUJLB01.1/dataset/CAUJLB01.1/cumulative.

The snail plot in Figure 2 provides a summary of the assembly statistics, indicating the distribution of scaffold lengths and other assembly metrics. Figure 3 shows the distribution of scaffolds by GC proportion and coverage. Figure 4 presents a cumulative assembly plot, with separate curves representing different scaffold subsets assigned to various phyla, illustrating the completeness of the assembly.

**Figure 5.**
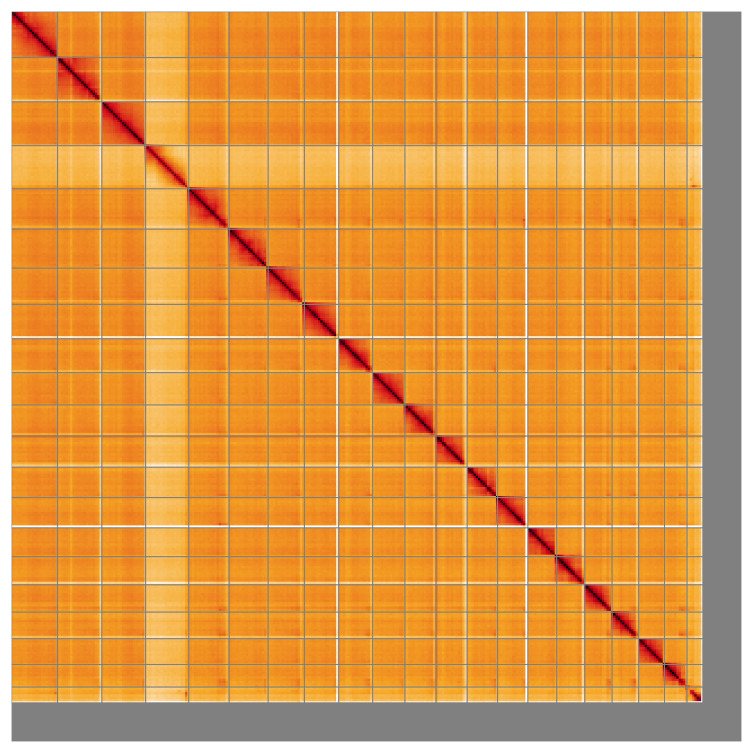
Genome assembly of *Neocrepidodera transversa* icNeoTran1.1: Hi-C contact map of the icNeoTran1.1 assembly, visualised using HiGlass. Chromosomes are shown in order of size from left to right and top to bottom. An interactive version of this figure may be viewed at https://genome-note-higlass.tol.sanger.ac.uk/l/?d=T8NOvPmyQdO0VPeCoN-Nlg.

Most of the assembly sequence (93.58%) was assigned to 21 chromosomal-level scaffolds, consisting of 20 autosomes and the X chromosome, which was assigned based on read coverage statistics. These chromosome-level scaffolds, confirmed by the Hi-C data, are named in order of size ( Figure 5; Table 3). Chromosome 20 is likely to be the Y chromosome, but this could not be confirmed.

**Table 3.** Chromosomal pseudomolecules in the genome assembly of *Neocrepidodera transversa*, icNeoTran1.

INSDC accession	Name	Length (Mb)	GC%
OY725336.1	1	41.73	30.0
OY725337.1	2	40.34	30.5
OY725338.1	3	39.71	29.5
OY725340.1	4	36.77	30.5
OY725341.1	5	35.43	30.0
OY725342.1	6	32.85	30.0
OY725343.1	7	31.18	29.5
OY725344.1	8	30.94	30.0
OY725345.1	9	29.28	30.0
OY725346.1	10	28.29	30.0
OY725347.1	11	28.28	30.5
OY725348.1	12	27.66	30.0
OY725349.1	13	27.43	30.0
OY725350.1	14	26.29	30.0
OY725351.1	15	25.5	29.0
OY725352.1	16	24.76	30.0
OY725353.1	17	24.22	30.0
OY725354.1	18	23.46	29.5
OY725355.1	19	20.55	30.0
OY725356.1	20	14.3	30.0
OY725339.1	X	39.23	29.5
OY725357.1	MT	0.02	22.5

While not fully phased, the assembly deposited is of one haplotype. Contigs corresponding to an alternate haplotype have also been deposited. The mitochondrial genome was also assembled and can be found as a contig within the multifasta file of the genome submission, and as a separate fasta file.

The final assembly has a Quality Value (QV) of 60.3. The primary assembly has a *k*-mer completeness of 75.41%, the alternate haplotype 68.25%, while the combined assemblies have a *k*-mer completeness of 98.05%. BUSCO (v5.4.3) analysis using the endopterygota_odb10 reference set ( *n* = 2,124) indicated a completeness score of 98.9% (single = 97.5%, duplicated = 1.4%). The assembly achieves the EBP reference standard of 6.C.60. Other quality metrics are given in Table 2.

## Genome annotation report

The *Neocrepidodera transversa* genome assembly (GCA_963243735.1) was annotated at the European Bioinformatics Institute (EBI) on Ensembl Rapid Release. The resulting annotation includes 22,975 transcribed mRNAs from 13,840 protein-coding and 1,881 non-coding genes ( Table 2; https://rapid.ensembl.org/Neocrepidodera_transversa_GCA_963243735.1/Info/Index). The average transcript length is 16,713.24. There are 1.46 coding transcripts per gene and 5.93 exons per transcript.

## Methods

### Sample acquisition and DNA barcoding

Specimens of *Neocrepidodera transversa* were collected from Hartslock Nature Reserve, England, United Kingdom (latitude 51.51, longitude –1.11) on 2021-07-29, using an aerial net. The specimens were collected by Ian Sims (the British Entomological and Natural History Society), identified by Michael Geiser (Natural History Museum), and preserved by dry freezing at –80 °C. One of the specimens (specimen ID NHMUK014536712, ToLID icNeoTran1) was used for DNA HiFi and Hi-C sequencing and another (specimen ID NHMUK014443414, ToLID icNeoTran2) was used for RNA sequencing.

The initial identification was verified by an additional DNA barcoding process according to the framework developed by Twyford *et al*. (2024). A small sample was dissected from the specimens and stored in ethanol, while the remaining parts were shipped on dry ice to the Wellcome Sanger Institute (WSI). The tissue was lysed, the COI marker region was amplified by PCR, and amplicons were sequenced and compared to the BOLD database, confirming the species identification ( Crowley *et al.*, 2023). Following whole genome sequence generation, the relevant DNA barcode region was also used alongside the initial barcoding data for sample tracking at the WSI ( Twyford *et al.*, 2024). The standard operating procedures for Darwin Tree of Life barcoding have been deposited on protocols.io ( Beasley *et al.*, 2023).

### Nucleic acid extraction

The workflow for high molecular weight (HMW) DNA extraction at the Wellcome Sanger Institute (WSI) Tree of Life Core Laboratory includes a sequence of procedures: sample preparation and homogenisation, DNA extraction, fragmentation and purification. Detailed protocols are available on protocols.io ( Denton *et al.*, 2023b).

The icNeoTran1 sample was prepared for DNA extraction by weighing and dissecting it on dry ice ( Jay *et al.*, 2023), and tissue from the abdomen was homogenised using a PowerMasher II tissue disruptor ( Denton *et al.*, 2023a).

HMW DNA was extracted in the WSI Scientific Operations core using the Automated MagAttract v2 protocol ( Oatley *et al.*, 2023). The DNA was sheared into an average fragment size of 12–20 kb in a Megaruptor 3 system ( Bates *et al.*, 2023). Sheared DNA was purified by solid-phase reversible immobilisation, using AMPure PB beads to eliminate shorter fragments and concentrate the DNA ( Strickland *et al.*, 2023). The concentration of the sheared and purified DNA was assessed using a Nanodrop spectrophotometer and Qubit Fluorometer using the Qubit dsDNA High Sensitivity Assay kit. Fragment size distribution was evaluated by running the sample on the FemtoPulse system.

RNA was extracted from whole organism tissue of icNeoTran2 in the Tree of Life Laboratory at the WSI using the RNA Extraction: Automated MagMax™ *mir*Vana protocol ( do Amaral *et al.*, 2023). The RNA concentration was assessed using a Nanodrop spectrophotometer and a Qubit Fluorometer using the Qubit RNA Broad-Range Assay kit. Analysis of the integrity of the RNA was done using the Agilent RNA 6000 Pico Kit and Eukaryotic Total RNA assay.

### Hi-C preparation

Tissue from the head and thorax of the icNeoTran1 sample was processed at the WSI Scientific Operations core, using the Arima-HiC v2 kit. Tissue (stored at –80 °C) was fixed, and the DNA crosslinked using a TC buffer with 22% formaldehyde. After crosslinking, the tissue was homogenised using the Diagnocine Power Masher-II and BioMasher-II tubes and pestles. Following the kit manufacturer's instructions, crosslinked DNA was digested using a restriction enzyme master mix. The 5’-overhangs were then filled in and labelled with biotinylated nucleotides and proximally ligated. An overnight incubation was carried out for enzymes to digest remaining proteins and for crosslinks to reverse. A clean up was performed with SPRIselect beads prior to library preparation.

### Library preparation and sequencing

Library preparation and sequencing were performed at the WSI Scientific Operations core. Pacific Biosciences HiFi circular consensus DNA sequencing libraries were prepared using the PacBio Express Template Preparation Kit v2.0 (Pacific Biosciences, California, USA) as per the manufacturer's instructions. The kit includes the reagents required for removal of single-strand overhangs, DNA damage repair, end repair/A-tailing, adapter ligation, and nuclease treatment. Library preparation also included a library purification step using AMPure PB beads (Pacific Biosciences, California, USA) and size selection step to remove templates shorter than 3 kb using AMPure PB modified SPRI. DNA concentration was quantified using the Qubit Fluorometer v2.0 and Qubit HS Assay Kit and the final library fragment size analysis was carried out using the Agilent Femto Pulse Automated Pulsed Field CE Instrument and gDNA 165kb gDNA and 55kb BAC analysis kit. Samples were sequenced using the Sequel IIe system (Pacific Biosciences, California, USA). The concentration of the library loaded onto the Sequel IIe was in the range 40–135 pM. The SMRT link software, a PacBio web-based end-to-end workflow manager, was used to set-up and monitor the run, as well as perform primary and secondary analysis of the data upon completion.

For Hi-C library preparation, DNA was fragmented to a size of 400 to 600 bp using a Covaris E220 sonicator. The DNA was then enriched, barcoded, and amplified using the NEBNext Ultra II DNA Library Prep Kit following manufacturers’ instructions. The Hi-C sequencing was performed using paired-end sequencing with a read length of 150 bp on an Illumina NovaSeq 6000 instrument.

Poly(A) RNA-Seq libraries were constructed using the NEB Ultra II RNA Library Prep kit, following the manufacturer’s instructions. RNA sequencing was performed on the Illumina NovaSeq 6000 instrument.

### Genome assembly, curation and evaluation

***Assembly***

The HiFi reads were first assembled using Hifiasm ( Cheng *et al.*, 2021) with the --primary option. Haplotypic duplications were identified and removed using purge_dups ( Guan *et al.*, 2020). The Hi-C reads were mapped to the primary contigs using bwa-mem2 ( Vasimuddin *et al.*, 2019). The contigs were further scaffolded using the provided Hi-C data ( Rao *et al.*, 2014) in YaHS ( Zhou *et al.*, 2023) using the --break option for handling potential misassemblies. The scaffolded assemblies were evaluated using Gfastats ( Formenti *et al.*, 2022), BUSCO ( Manni *et al.*, 2021) and MERQURY.FK ( Rhie *et al.*, 2020).

The mitochondrial genome was assembled using MitoHiFi ( Uliano-Silva *et al.*, 2023), which runs MitoFinder ( Allio *et al.*, 2020) and uses these annotations to select the final mitochondrial contig and to ensure the general quality of the sequence.

***Assembly curation***

The assembly was decontaminated using the Assembly Screen for Cobionts and Contaminants (ASCC) pipeline (article in preparation). Manual curation was primarily conducted using PretextView ( Harry, 2022), with additional insights provided by JBrowse2 ( Diesh *et al.*, 2023) and HiGlass ( Kerpedjiev *et al.*, 2018). Scaffolds were visually inspected and corrected as described by Howe *et al*. (2021). Any identified contamination, missed joins, and mis-joins were corrected, and duplicate sequences were tagged and removed. The curation process is documented at https://gitlab.com/wtsi-grit/rapid-curation (article in preparation).

***Assembly quality assessment***

The Merqury.FK tool ( Rhie *et al.*, 2020), run in a Singularity container ( Kurtzer *et al.*, 2017), was used to evaluate *k*-mer completeness and assembly quality for the primary and alternate haplotypes using the *k*-mer databases ( *k* = 31) that were computed prior to genome assembly. The analysis outputs included assembly QV scores and completeness statistics. The genome was also analysed within the BlobToolKit environment ( Challis *et al.*, 2020) and BUSCO scores ( Manni *et al.*, 2021) were calculated.

A Hi-C contact map was produced for the final version of the assembly. The Hi-C reads were aligned using bwa-mem2 ( Vasimuddin *et al.*, 2019) and the alignment files were combined using SAMtools ( Danecek *et al.*, 2021). The Hi-C alignments were converted into a contact map using BEDTools ( Quinlan & Hall, 2010) and the Cooler tool suite ( Abdennur & Mirny, 2020). The contact map was visualised in HiGlass ( Kerpedjiev *et al.*, 2018).

Table 4 contains a list of relevant software tool versions and sources.

**Table 4.** Software tools: versions and sources.

Software tool	Version	Source
BEDTools	2.30.0	https://github.com/arq5x/bedtools2
BLAST	2.14.0	ftp://ftp.ncbi.nlm.nih.gov/blast/executables/blast+/
BlobToolKit	4.3.7	https://github.com/blobtoolkit/blobtoolkit
BUSCO	5.4.3 and 5.5.0	https://gitlab.com/ezlab/busco
bwa-mem2	2.2.1	https://github.com/bwa-mem2/bwa-mem2
Cooler	0.8.11	https://github.com/open2c/cooler
DIAMOND	2.1.8	https://github.com/bbuchfink/diamond
fasta_windows	0.2.4	https://github.com/tolkit/fasta_windows
FastK	427104ea91c78c3b8b8b49f1a7d6bbeaa869ba1c	https://github.com/thegenemyers/FASTK
Gfastats	1.3.6	https://github.com/vgl-hub/gfastats
GoaT CLI	0.2.5	https://github.com/genomehubs/goat-cli
Hifiasm	0.16.1-r375	https://github.com/chhylp123/hifiasm
HiGlass	44086069ee7d4d3f6f3f0012569789ec138f42b84a a44357826c0b6753eb28de	https://github.com/higlass/higlass
Merqury.FK	d00d98157618f4e8d1a9190026b19b471055b22e	https://github.com/thegenemyers/MERQURY.FK
MitoHiFi	2	https://github.com/marcelauliano/MitoHiFi
MultiQC	1.14, 1.17, and 1.18	https://github.com/MultiQC/MultiQC
NCBI Datasets	15.12.0	https://github.com/ncbi/datasets
Nextflow	23.04.0-5857	https://github.com/nextflow-io/nextflow
PretextView	0.2.5	https://github.com/sanger-tol/PretextView
purge_dups	1.2.3	https://github.com/dfguan/purge_dups
samtools	1.16.1, 1.17, and 1.18	https://github.com/samtools/samtools
sanger-tol/ascc	-	https://github.com/sanger-tol/ascc
Seqtk	1.3	https://github.com/lh3/seqtk
Singularity	3.9.0	https://github.com/sylabs/singularity
TreeVal	1.0.0	https://github.com/sanger-tol/treeval
YaHS	1.2a	https://github.com/c-zhou/yahs

### Genome annotation

The Ensembl Genebuild annotation system ( Aken *et al.*, 2016) was used to generate annotation for the *Neocrepidodera transversa* assembly (GCA_963243735.1) in Ensembl Rapid Release at the EBI. Annotation was created primarily through alignment of transcriptomic data to the genome, with gap filling via protein-to-genome alignments of a select set of proteins from UniProt ( UniProt Consortium, 2019).

### Wellcome Sanger Institute – Legal and Governance

The materials that have contributed to this genome note have been supplied by a Darwin Tree of Life Partner. The submission of materials by a Darwin Tree of Life Partner is subject to the **‘Darwin Tree of Life Project Sampling Code of Practice’**, which can be found in full on the Darwin Tree of Life website here. By agreeing with and signing up to the Sampling Code of Practice, the Darwin Tree of Life Partner agrees they will meet the legal and ethical requirements and standards set out within this document in respect of all samples acquired for, and supplied to, the Darwin Tree of Life Project.

Further, the Wellcome Sanger Institute employs a process whereby due diligence is carried out proportionate to the nature of the materials themselves, and the circumstances under which they have been/are to be collected and provided for use. The purpose of this is to address and mitigate any potential legal and/or ethical implications of receipt and use of the materials as part of the research project, and to ensure that in doing so we align with best practice wherever possible. The overarching areas of consideration are:

•     Ethical review of provenance and sourcing of the material

•     Legality of collection, transfer and use (national and international)

Each transfer of samples is further undertaken according to a Research Collaboration Agreement or Material Transfer Agreement entered into by the Darwin Tree of Life Partner, Genome Research Limited (operating as the Wellcome Sanger Institute), and in some circumstances other Darwin Tree of Life collaborators.

## Data Availability

European Nucleotide Archive: Neocrepidodera transversa. Accession number PRJEB59946; https://identifiers.org/ena.embl/PRJEB59946. The genome sequence is released openly for reuse. The *Neocrepidodera transversa* genome sequencing initiative is part of the Darwin Tree of Life (DToL) project. All raw sequence data and the assembly have been deposited in INSDC databases. Raw data and assembly accession identifiers are reported in Table 1 and Table 2.

## Author information

Members of the Natural History Museum Genome Acquisition Lab are listed here: https://doi.org/10.5281/zenodo.12159242.

Members of the Darwin Tree of Life Barcoding collective are listed here: https://doi.org/10.5281/zenodo.12158331.

Members of the Wellcome Sanger Institute Tree of Life Management, Samples and Laboratory team are listed here: https://doi.org/10.5281/zenodo.12162482.

Members of Wellcome Sanger Institute Scientific Operations: Sequencing Operations are listed here: https://doi.org/10.5281/zenodo.12165051.

Members of the Wellcome Sanger Institute Tree of Life Core Informatics team are listed here: https://doi.org/10.5281/zenodo.12160324.

Members of the Tree of Life Core Informatics collective are listed here: https://doi.org/10.5281/zenodo.12205391.

Members of the Darwin Tree of Life Consortium are listed here: https://doi.org/10.5281/zenodo.4783558.
