# Supplementary material for: The genome sequence of a flea beetle, Neocrepidodera transversa (Marsham, 1802)
Source: Wellcome Open Res. 2025 Feb 10;10:62. [Version 1] doi: 10.12688/wellcomeopenres.23697.1 (PMC11897695; doi:10.12688/wellcomeopenres.23697.1)
Supplement: Peer review history

# Reviewer report: Reviewer response for version 1

**DOI:** 10.21956/wellcomeopenres.26143.r118460

**Published:** 2025-03-11

**Authors:** Doga Cedden

**Affiliations:** 1 University of Göttingen, Göttingen, Germany

Geiser *et al*. reports a chromosomally complete genome assembly of sufficient quality from a flea beetle, *Neocrepidodera transversa*. The background information is appropriate and adequate. The genome sequence is accessible via the provided link. I would suggest including a scale bar in Fig. 1. As the specimen is no longer available, at least the size of the paper label shown in Fig. 1 could be provided in the caption.

Are sufficient details of methods and materials provided to allow replication by others?

Yes

Is the rationale for creating the dataset(s) clearly described?

Yes

Are the datasets clearly presented in a useable and accessible format?

Yes

Are the protocols appropriate and is the work technically sound?

Yes

Reviewer Expertise:

I work on coleopteran pests using RNAi and RNA-seq.

I confirm that I have read this submission and believe that I have an appropriate level of expertise to confirm that it is of an acceptable scientific standard.

# Reviewer report: Reviewer response for version 1

**DOI:** 10.21956/wellcomeopenres.26143.r118457

**Published:** 2025-02-26

**Authors:** Joaquín Ortego

**Affiliations:** 1 Estación Biológica de Doñana, Seville, Spain

In this manuscript, the authors present a nuclear and mitochondrial genome assembly for the leaf beetle *Neocrepidodera transversa*. They combined long-read sequencing (PacBio HiFi) and Hi-C technology to produce a chromosome-level genome assembly for their target species. The assembly was 671.30 Mb in length, with an estimated 34-fold coverage, and was organized into 21 chromosomal pseudomolecules, with a BUSCO completeness of 98.9%. The authors have also annotated the genome, identifying over 13,000 protein-coding genes. I found the study to be well-executed, providing all the necessary methodological details. Overall, I believe this report presents a high-quality genome assembly and is ready for indexing.

Are sufficient details of methods and materials provided to allow replication by others?

Yes

Is the rationale for creating the dataset(s) clearly described?

Yes

Are the datasets clearly presented in a useable and accessible format?

Yes

Are the protocols appropriate and is the work technically sound?

Yes

Reviewer Expertise:

Area of research: Molecular Ecology; Population genetics; Phylogeography; Biogeography; Conservation Genetics. I have limited experience with genome sequencing and assembling.

I confirm that I have read this submission and believe that I have an appropriate level of expertise to confirm that it is of an acceptable scientific standard.
